# Fabry disease – a multisystemic disease with gastrointestinal manifestations

**DOI:** 10.1080/19490976.2022.2027852

**Published:** 2022-01-28

**Authors:** Malte Lenders, Eva Brand

**Affiliations:** Internal Medicine D, Interdisciplinary Fabry Center Münster (IFAZ), University Hospital Münster, Münster, Germany

**Keywords:** Diarrhea, Fabry disease, globotriaosylceramide, inflammatory bowel disease, treatment

## Abstract

Nonspecific gastrointestinal (GI) symptoms, such as postprandial cramping pain, diarrhea, nausea and vomiting are typical symptoms for irritable bowel syndrome or inflammatory bowel disease, but may also be the first symptoms of Fabry disease (FD). This review focus on GI manifestations in FD, by providing an overview of symptoms, a proper diagnosis, an appropriate management by FD-specific and concomitant medications and lifestyle interventions. We provide comprehensive literature-based data combined with personal experience in the management of FD patients. Since FD is rare and the clinical phenotype is heterogeneous, affected patients are often misdiagnosed. Consequently, physicians should consider FD as a possible differential diagnosis when assessing unspecific GI symptoms. Improved diagnostic tools, such as a modified GI symptom assessment scale can facilitate the diagnosis of FD in patients with GI symptoms of unknown cause and thus enable the timely initiation of a disease-specific therapy. Expansive intravenous enzyme replacement therapy with α-galactosidase A or oral chaperone therapy for patients with amenable mutations improve the disease burden including GI symptoms, but a timely start of therapy is crucial for the prognosis. A special diet low in fermentable oligosaccharides, disaccharides, monosaccharides and polyols (FODMAP) or pro- and prebiotics might improve FD-typical GI symptoms. Furthermore, preliminary success was reported with the oral administration of α-galactosidase A. In addition to a timely initiation of FD-specific therapy, affected patients with GI symptoms might benefit from a FODMAP-low diet, pro- and prebiotics and/or low-cost oral substitution with AGAL to support digestion and reduce dysbiosis.

## Introduction

Gastrointestinal (GI) symptoms of unknown causes often represent a time-consuming clinical-diagnostic challenge not only for gastroenterologists but also for general practitioners, internists, and pediatricians. Differential diagnoses include common diseases such as irritable bowel syndrome (IBS) and inflammatory bowel disease (IBD), but also more rarely hereditary metabolic diseases such as Fabry disease (FD).

FD (Online Mendelian Inheritance in Man [OMIM] #301,500) is an X-chromosomal-linked inborn error due to various mutations within the *α-galactosidase A* (*GLA*/AGAL) gene, resulting in a deficient enzymatic AGAL activity. The worldwide incidence of FD has been estimated at 1 in 40,000 to 1 in 117,000 live male births.^1^ However, newborn screenings showed that the incidence of FD is more common, reporting 1:3,200 with the inclusion of late-onset and milder *GLA* variants.^2,3^ To date, more than 1,000 *GLA* mutations have been identified, according to the Human GeneMutation Database (http://www.hgmd.cf.ac.uk/), resulting in a wide spectrum of clinical symptoms and manifestations.^4^ Fabry-specific manifestations are a consequence of systemic accumulation of glycolipids (globotriaosylceramide [Gb_3_]) mainly in the vascular endothelium, particularly in the kidneys, heart, nervous system, and skin alone or in combination with damage from deposits in almost all cell types (endothelial cells, nerve cells, muscle cells, among others).^5^ The progressive Gb_3_ accumulation is accompanied by a high risk of early onset of stroke, life-threatening arrhythmia, myocardial infarction, or cardiac and renal failures. The deacylated and soluble form of Gb_3_, globotriaosylsphingosine (lyso-Gb_3_), seems to be a reliable biomarker for both disease progression and therapy efficacy, and is measured in blood plasma as well as urine.^6–12^ In addition to renal, cardiac, and neurological manifestations, nonspecific GI symptoms including abdominal pain, diarrhea, constipation, bloating, nausea, and vomiting occur frequently and already appear in childhood. These symptoms often lead to a significantly reduced quality of life. Since FD is rare and the clinical phenotype is heterogeneous, affected patients are often misdiagnosed and therefore the diagnosis is delayed. In patients with milder or late-onset phenotypes, nonspecific GI symptoms can appear later in life (compared to classic phenotypes), leading to a diagnostic challenge, especially in those without a family history of FD.^13^

FD is currently treatable by enzyme replacement therapy (ERT; agalsidase-alfa, 0.2 mg/kg body weight [b.w.] every other week [e.o.w.], and agalsidase-beta, 1.0 mg/kg b.w. e.o.w, intravenously)^14,15^ or chaperone therapy (oral migalastat 123 mg every other day)^16^ with estimated annual costs of ~250.000€ per year and patient in Europe. Safety and efficacy of both approved therapy strategies (ERT and chaperone) were repeatedly demonstrated, although their impact on disease progression is affected by various conditions including age and disease load at therapy initiation, dosage, sex, neutralizing anti-drug antibodies against ERT and type of *GLA* mutation. Since therapy is expensive, consensus recommendations for the initiation and cessation of ERT in patients with FD are published.^17,18^ Depending on sex and the extent of organ manifestation at the heart, kidney, and brain, Class I/II recommendations for an FD-specific treatment are proposed, clearly suggesting a beneficial impact on disease progression.^17^ In this respect, a Class I recommendation (FD-specific treatment is recommended/is indicated) is provided, if evidence and/or general agreement is present that a given treatment or procedure is beneficial, useful, and effective.^17^ A Class IIA recommendation (FD-specific treatment should be considered) is provided, if the weight of evidence or expert opinion is in favor of usefulness and efficacy. In addition, if the usefulness or efficacy of FD-specific treatment is less well established by evidence or expert opinion, a Class IIB recommendation (FD-specific treatment may be considered) is suggested. Treatment may be considered in patients with GI symptoms (Class IIA if <16 years of age, Class IIB if >16 years of age).^17^ Since GI symptoms severely reduce the quality of life, this may result in a therapeutic dilemma.

The aim of this review is to draw more attention to GI manifestations by providing an overview of GI symptoms in FD, a proper diagnosis and the pathophysiology with underlying mechanisms. We also provide an overview for an appropriate management and therapy strategies by FD-specific treatments, including concomitant medications and suitable lifestyle interventions, such as potential cost-effective diets and nutritional supplements, which might positively affect GI symptoms and increase patients’ quality of life.

## Clinical case

For a better understanding of the clinical symptoms and complexity of FD diagnosis, the following clinical case may be helpful. A 38-year-old male patient presented to our Fabry center: during a dermatological treatment of a lichen sclerosus in the genital area, multiple angiokeratomas periumbilical and in the swim trunk area were noticeable, suggesting the differential diagnosis of FD. Since the age of eight, the patient suffered from mostly postprandial gastrointestinal cramping pain, flatulence, and diarrhea. He avoided food intake in the morning and at noon in order to be able to pursue his professional activity without gastrointestinal pain. There was underweight (body mass index 19 kg/m^2^). Regarding the gastrointestinal complaints, multiple blood and stool diagnostics (*Salmonella, Shigella, Campylobacter jejuni/coli, Yersinia*), examinations regarding lactose and fructose intolerance, abdominal ultrasonography, several gastroscopies (no evidence of *Helicobacter pylori*) and coloscopies were without any indicative findings. The complaints were misdiagnosed as irritable bowel syndrome. Our diagnostic workup confirmed the suspected clinical diagnosis of FD with a markedly decreased enzymatic AGAL activity (<2.8 µmol/l/h; reference: ≥15.3 µmol/l/h), an increased lyso-Gb_3_ (81 ng/ml, reference ≤1.8 ng/ml), and evidence of a pathogenic AGAL mutation (p.Arg112 Cys, hemizygous). Echocardiography revealed incipient left ventricular hypertrophy (interventricular septum and posterior wall 12 mm, mild diastolic dysfunction). Cardiac MRI showed late enhancement at the lateral/posterior wall. The onset of Fabry nephropathy was manifested by albuminuria (albumin/creatinine 240 mg/dl). The patient reported FD-typical anhidrosis and intermittent tinnitus. After initiation of FD-specific therapy with agalsidase-alfa (0.2 mg/kg b.w., e.o.w.) the gastrointestinal symptoms showed marked improvement in terms of frequency and intensity of occurrence; plasma lyso-Gb_3_ decreased below 30 ng/ml. In the course of a family screening, the mutation was also identified in the patient’s sister and a niece, without current indication for therapy.

## Gastrointestinal symptoms in Fabry disease

**Figure 1.**
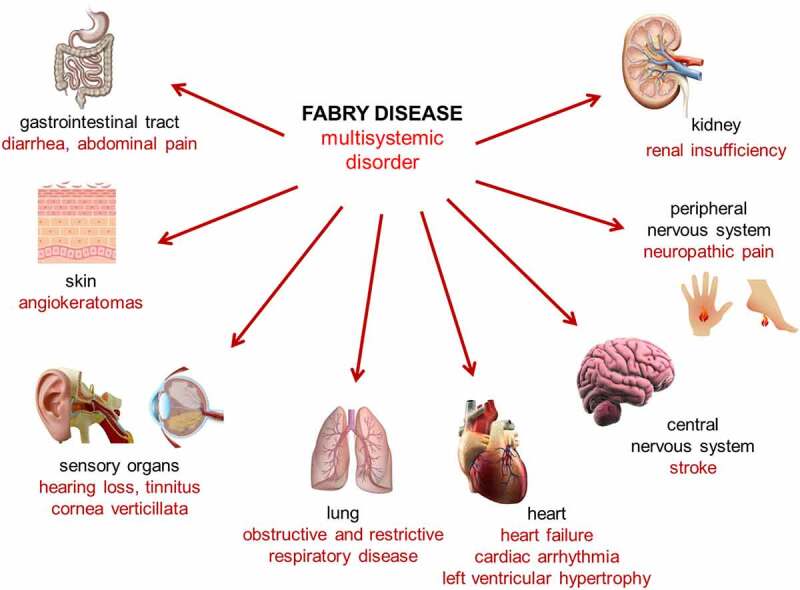
Fabry disease is a multisystemic disorder.

FD is a multisystemic disorder (Figure 1). GI symptoms belong to the first manifestations already in affected pediatric FD patients.^19^ Abdominal pain and diarrhea are the most common symptoms, followed by constipation, nausea, and vomiting.^13,20–23^ In detail, registry data from the Fabry Outcome Survey (FOS) based on 1,453 patients reported a prevalence of 51% for GI symptoms^24^ mainly due to abdominal pain and diarrhea.^20^ Abdominal pain is the most frequently reported symptom in affected patients and includes the appearance of colic with pain in the mid- or lower abdomen, bloating, cramping, or mid-abdominal discomfort.^25,26^ Since these symptoms may increase during or after meals or are triggered by stress, it is conceivable that many FD patients are reluctant to food intake, which may result in lower body weight. However, this seems to be limited to patients with very severe symptoms, since most studies and reports did not show differences in body mass index between patients with and without GI symptoms.^2^ Frequency and severity of diarrhea as the second most GI symptom is more diverse. According to the FOS registry, 20% of FD patients reported diarrhea, which was more common in males (26%) than in females (17%), and very frequent in children (25%).^20,27^ However, the real frequency in classical FD patients seems to be much higher, since the reported frequency in females with FD manifestations justifying ERT from the Fabry Registry is reported as 39%.^23^

Diarrhea often appears postprandial (after food intake) and can occur up to 12 times a day, significantly reducing the quality of life of affected patients.^25,28^ Further symptoms include the appearance of nausea, vomiting (both more frequent in children) and constipation, while females seem to be more often affected by the latter than males.^17^ Mainly in adult patients, the presence of gastritis, hemorrhoids, chronic intestinal pseudo-obstruction, diverticular disease, and bowel ischemia was also reported.^20,25,29–33^

## Pathophysiology and potential mechanisms

Due to the complexity, the pathology of GI symptoms in FD is not very well understood, so far. Currently, five important non-exclusive mechanisms are supposed to be involved: i) dysfunction of the autonomic nervous system, adversely affecting gut motility, ii) vasculopathy, controlling GI blood circulation, iii) tissue inflammation, triggered by (lyso)-Gb_3_ accumulation, iv) dysbiosis and bacterial overgrowth, v) lack of AGAL might result in a maldigestion of nutrition within the gut.^13,26,33,34^

It is supposed that these processes may lead to a rapid intestinal passage as well as reduced intestinal peristalsis, intestinal stasis, pancreatic insufficiency, gastroparesis, an ischemic or neuropathic damage, and disturbed gut microflora balance, leading to GI symptoms and manifestations of FD.^13^

Globotriaosylceramide (Gb_3_) accumulation in muscle cells, endothelial cells, and nerve cells (submucosal and myenteric plexus)^29,32,35–37^ leads to enlargement of the villi, vasculopathy, and altered conduction of the enteric nerve signal, resulting in gastric dysfunction, including food malabsorption by the villi and postprandial abdominal pain, early satiety through food, nausea, and diarrhea, potentially leading to an imbalance of the gut microflora.^13,26,29,32,36^

Dysmotility-induced high intraluminal pressure can lead to diverticula in the duodenum, jejunum, and colon with sometimes fatal consequences for affected patients.^30,32,35,36^ Pancreatic dysfunction may cause post-prandial diarrhea, especially after consumption of fatty meals.^38^

FD is a small vessel disease due to the accumulation of Gb_3_ within endothelial cells and smooth muscle cells, leading to endothelial dysfunction. The resulting vasculopathy in small vessels of the GI compartment may also lead to ischemic changes in the abdominal vasculature and could be responsible for observed GI symptoms including abdominal pain, impaired food absorption, and general inflammatory processes, too.^26,30,35,36,39^

Recent studies have demonstrated an elevated inflammatory profile in patients with FD,^40–43^ but literature dealing with specific anti-inflammatory medication in patients with FD is scarce, and no specific recommendations for classes of anti-inflammation agents are currently suggested. In this respect, at least cell culture models for FD demonstrated an anti-inflammatory effect of pentosan polysulfate.^44^ However, non-steroidal anti-inflammatory drugs should be used with caution in patients with Fabry nephropathy, due to their potential nephrotoxicity.

Furthermore, a recent preliminary study also reported that lyso-Gb_3_ modifies the biology of the gut microbiome, favoring the production of biofilms and altering the composition and short-chain fatty-acid profile of the gut microbiota.^45^ Finally, an increased accumulation of CD77 (Gb_3_) on epithelial surface might also facilitate uptake and internalization of bacteriological enterotoxins, such as Shiga toxins as demonstrated for endothelial cells,^46^ resulting in a higher risk for bacterial-induced cell damage. Also, in this respect, the Gb_3_ derivate isoglobotriaosylceramide was identified as a ligand for CD1d, which is involved in the activation of invariant natural killer T (iNKT) cells.^47^ The CD1d-dependent iNKT cell activation is part of the host defense in the intestine. Hence, it is likely that activation of iNKT cells leads to an altered (and thus increased) inflammatory state in affected FD patients, triggering GI symptoms.^48^

In addition to a poor intestinal motility, recent studies suggest that lyso-Gb_3_ directly affects microbiotical growth,^45^ which might eventually lead to dysbiosis, an imbalance of the intestinal flora. Dysbiosis is associated with an increased pro-inflammatory immune response, due to an abnormal proliferation of immune cells and increases the production of pro-inflammatory compounds, such as lipopolysaccharides.^49^ Gut dysbiosis furthermore impairs the energy supply to the colonic epithelium and increases epithelial permeability, leading to a “leaky gut.”^50,51^ In this respect, lyso-Gb_3_ increases the biofilm-forming capacity of several individual bacteria, including *Bacteroides fragilis*.^45^ In detail, lyso-Gb_3_ also modifies the bacterial composition of the human colon microbiota suspension, increasing bacterial counts of *B. fragilis*, and modified the formation of short-chain fatty acids, leading to a striking decrease in butyrate concentration.^45^ In inflammatory bowel disease, dysbiosis can result in decreased energy procurement and also inflammation,^52^ which might also be true for FD. A dysbiosis in FD patients with renal impairment might further be triggered by an increased urea secretion into the digestive system, contributing to circulating uremic toxins, systemic inflammation, oxidative stress, cardiovascular events, and other complications as observed in patients with end-stage renal disease (ESRD).^53^

GI symptoms in patients with FD might also be due to the lack of a proper galacto-oligosaccharide digestion within the intestine.^13,54^ In this respect, orally delivered AGAL digests and breaks down complex galacto-oligosaccharides (such as raffinose, stachyose, or verbascose), a process that probably reduces bowel symptoms including abdominal bloating and diarrhea by decreasing the colonic fermentation and thus gas production in patients with IBS.^55^

## Diagnosis of Fabry disease

Due to the variable clinical presentation, patients with FD often remain undiagnosed for many years. Delay in diagnosis of up to 20 years between the first symptoms and confirmed diagnosis is common.^25,56^ In patients with a long-term history of unexplained GI symptoms including postprandial abdominal pain, diarrhea, early satiety, gastroparesis, or chronical intestinal pseudo-obstruction FD should be considered for diagnosis.^13,26^ Furthermore, grouped angiokeratomas in the “swim trunks area” or umbilical could indicate FD. The presence of an ocular sign such as cornea verticillata, requiring a simple slit-lamp examination by an ophthalmologist, is also typical for a classical FD. Abnormal sweating (often hypohidrosis, occasionally hyperhidrosis), neuropathic pain in hands and feeds triggered by temperature changes or fever (especially in the childhood) are typical symptoms of FD, too. In males, the determination of AGAL activity in blood leukocytes is the gold standard for a confirmation of the diagnosis. A pathologically low AGAL activity indicates the presence of FD.^17^ Subsequently, a genotyping should also be performed to determine the type of mutation, which is especially important for further FD-specific treatment options, such as chaperone therapy. In females, a molecular genetic testing with detection of a disease-causing mutation of the *GLA* gene is necessary to confirm the diagnosis, since females often have AGAL activities within the reference range. As a biomarker (marker of disease burden), pathologically elevated lyso-Gb_3_ in plasma (or urine) will contribute to improve diagnosis and monitoring.^9,10^ Tissue biopsies may support diagnosis in uncertain cases, if multi-lamellar myelin bodies (so-called “zebra bodies”) are detected via electron microscopy. Although these “zebra bodies” are not pathognomonic of FD,^57^ their detection in relevant tissues obtained in the clinical context will suggest the diagnosis of FD and is recommended as the gold standard for the differential diagnosis of FD in adult subjects.^58^ Prenatal diagnosis can be performed by measuring AGAL activity in chorionic villi or cultured amniotic cells and, in the case of a mutation known in the family, by molecular genetic methods. Due to the multisystemic symptoms and manifestations of FD, clinicians should also evaluate if the patient suffers from additional manifestations such as renal or cardiac abnormalities, including loss of renal function (i.e. loss of eGFR, albuminuria/proteinuria), unexplained left ventricular hypertrophy, heart failure, and/or cardiac arrhythmia. A subsequent family history of such symptoms (or FD itself) is also highly warranted. The combination of GI symptoms and additional FD-typical manifestations increases the pretest probability of identifying an FD patient.

## Differential diagnosis

Due to the varied and nonspecific GI symptoms, it is important to exclude other underlying diseases to avoid misdiagnosis in already diagnosed FD patients and those who have not yet been diagnosed. Thus, potential differential diagnoses of adult patients with nonspecific gastrointestinal symptoms should include the following diseases:^13^ IBS (particularly diarrhea-predominant IBS), recurrent abdominal pain syndrome, chronic inflammatory bowel disease, appendicitis, Whipple’s disease, dermatomyositis, diverticular disease, somatoform disorder, Crohn’s disease, celiac disease, colon cancer, FD, mitochondrial diseases, transthyretin-related familial amyloid polyneuropathy. If no other reason for the observed symptoms is detected during endoscopy (gastroscopy, colonoscopy), biopsies with appropriate anti-Gb_3_ immunostaining^34^ or electron microscopy^56^ can demonstrate the presence of Gb_3_ deposits in epithelial gut cells of classical FD patients underlining the diagnosis of FD. In this respect, the requesting physician needs to include the differential diagnosis of FD in order to send an appropriately prepared biopsy for diagnosis, since routine biopsy will not detect FD.

## Treatment of gastrointestinal symptoms in Fabry disease

FD is treatable by enzyme replacement therapy (ERT)^14,15^ or chaperone therapy.^16^ According to current European recommendations for patients with FD, the presence of GI symptoms that are not successfully treated with a symptomatic therapy justifies the initiation of an FD-specific therapy.^17^ Both cost-intensive ERT and chaperone strategies are reported to significantly improve FD-typical GI symptoms, such as diarrhea and abdominal pain. However, the impact of ERT on GI symptoms is heterogeneous, which is also represented by the class of recommendations (Class IIA/B).^17^ In addition, the socio-economic burden of expensive therapies, such as ERT and chaperone therapy (~250,000 €/year per patient) is also a matter of debate, especially if GI symptoms are the only detected manifestations in an affected patient. A general overview of the currently approved and potential future FD-specific treatments is provided in Table 1.

**Table 1.** Overview of current approved and potential future treatment approaches for Fabry disease

Approved				
Treatment	Compound	Application	Cycle	Aim
enzyme replacement therapy	agalsidase-alfa, agalsidase-beta	intravenous	every other week	replacement of deficient AGAL
chaperone	migalastat	oral	every other day	increase of endogenous AGAL activity
**Non-approved**				
enzyme replacement therapy	pegunigalsidase-alfa, mossAGAL	intravenous	tba	replacement of deficient AGAL
substrate reduction therapy	venglustat, lucerastat	oral	every day	reduction of AGAL substrate
gene therapy	AVRO RD-01, ST920, FLT190, 4D-310	lentiviral-, adenoviral-mediated	tba	genomic insertion of functional AGAL in certain cells (hPSC-, hepatocyte-or cardiomyocyte-targeted)

AGAL: α-galactosidase A, tba: to be assessed.

### Impact of ERT on GI symptoms in FD

Treatment with either agalsidase-alfa (0.2 mg/kg b.w. e.o.w. intravenously; Replagal, Takeda) or agalsidase-beta (1.0 mg/kg b.w. e.o.w. intravenously; Fabrazyme, Sanofi Genzyme) was repeatedly reported to decrease frequencies of abdominal pain in affected adult and adolescent patients.^20,21,23,32,59–63^ Within the same studies, improvement of diarrhea,^21,23,32,59,62^ nausea and vomiting,^21,60,62^ and constipation^21,62^ were observed. In addition, the dose or compound might affect the outcome for GI symptoms. In this respect, patients who received a reduced dose of agalsidase-beta (0.3 to 0.5 mg/kg b.w. e.o.w. intravenously) or those who were switched from agalsidase-beta (1.0 mg/kg b.w.) to agalsidase-alfa (0.2 mg/kg b.w.) during the worldwide shortage of agalsidase-beta reported an increase of gastrointestinal pain.^64^ However, not all patients benefit from ERT, and GI symptoms are sometimes quite pronounced and relevant to daily life in ERT-treated patients, significantly reducing quality of life.

### Impact of pharmaceutical chaperone on GI symptoms in FD

In addition to ERT, a second treatment option based on a pharmaceutical chaperone (oral migalastat 123 mg every other day, 1-deoxygalactonojirimycin (DGJ); Galafold, Amicus Therapeutics) is approved in Europe since May 2016, in Canada since September 2017, in Japan since March 2018 and in the United States since August 2018 for long-term treatment of FD in adults (≥18 years of age in the United States and Canada, ≥16 years in Japan, and ≥12 years in Europe) for patients with an amenable mutation and an estimated glomerular filtration rate (eGFR) ≥30 ml/minute per 1.73 m^2^. Amenability, which means the response in terms of increasing enzymatic activities of an AGAL mutation to migalastat, is currently tested in a cell-culture-based good laboratory practice (GLP)-assay.^65^ Although approved since 2016, data on the impact of migalastat on GI symptoms are still scarce. However, in classical previously ERT-untreated males with amenable mutations, an AGAL activity <3% of normal values and a multi-organ system involvement, migalastat treatment resulted in a significant reduction of GI symptoms measured via the Gastrointestinal Symptom Rating Scale (GSRS) after 24 months.^66^ In patients previously treated with ERT, migalastat led to an improvement in diarrhea based on the Minimum Clinically Important Difference (MCID) score compared to those receiving a placebo.^67^

Despite FD-specific therapies with ERT or pharmaceutical chaperone, the impact of these drugs on GI symptoms is heterogeneous, probably due to the complexity of FD-related pathogenesis and mechanisms involved. Another reason is that GI symptoms that are pathogenically unrelated to FD cannot be improved by FD-specific treatments.

### Non FD-specific symptomatic treatment

Some major GI symptoms in patients with FD can be treated with various concomitant drugs that are symptomatically effective. Patients with acute diarrhea can be treated with classical anti-diarrhea medication such as loperamid. By contrast, patients suffering from gastroparesis can benefit from treatments with pro-motility agents, such as metoclopramide, which increase the contractile force and accelerate intraluminal transit.^68^ Patients suffering from upper GI symptoms may benefit from proton pump inhibitors (e.g. omeprazole) or ondansetron if nausea is present.^34^ Medication against bloating and flatulence may include the administration of simethicone, which eliminates and prevents foam formation.^69,70^ Furthermore, linaclotide, which is an oligo-peptide agonist of guanylate cyclase 2C is used to treat IBS with constipation and chronic constipation with unknown cause.^71,72^ The antispasmodic dicyclomine, which blocks the action of acetylcholine on cholinergic receptors in smooth muscles in the GI tract, is used to treat spasms of the intestine in IBS,^73^ and might also be of relevance, although it should be used with caution, especially in patients with any unstable cardiac condition.

However, most of these medications are not suitable for long-term use due to side effects. Hence, non-drug management approaches including dietary modifications such as for patients with IBS are probably indicated, but require a therapy-adherent patient.

### Nutritional and dietary supplemental effects on GI symptoms in FD

The effect of an AGAL deficiency on nutrition digestion has not been analyzed so far. To the best of our knowledge, no specific dietary interventions on FD patients have been reported, and recommendations are the same for IBS patients.^13,74^

**Figure 2.**
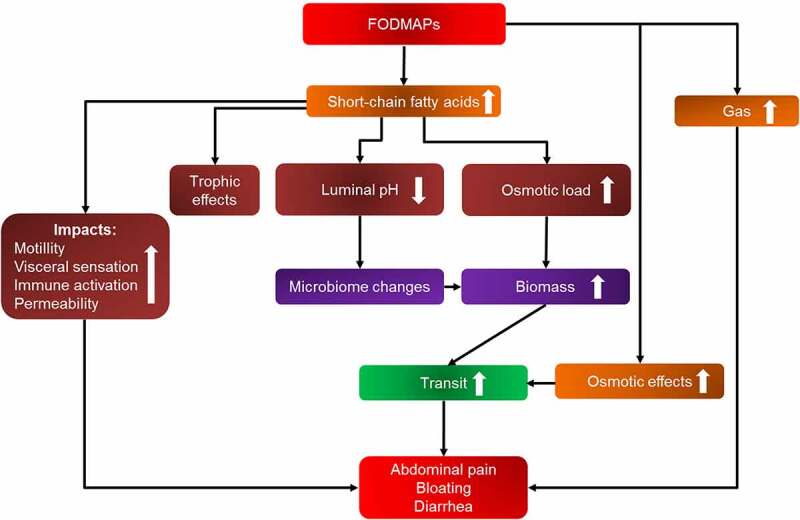
**Overview of potential mechanisms how FODMAP-rich nutrition may cause GI symptoms**. Abbreviations: FODMAPs, fermentable oligosaccharides, disaccharides, monosaccharides, and polyols; GI, gastrointestinal.

In general, appropriate recommendations for patients with IBS include a reduced intake of caffeine, alcohol, fat, and spicy food. Since irregular food intake deteriorates colonic motility and worsens IBS,^75^ FD patients should also follow a regular food intake (breakfast, lunch, dinner) and avoid larger meals. In addition, a special dietary seems advantageous. Recent studies demonstrated that patients with IBS benefit from a diet low in fermentable oligosaccharides, disaccharides, monosaccharides, and polyols (FODMAP), since short-chain fermentable carbohydrates exert osmotic effects that draw water into the intestinal and/or colonic lumen and FODMAPs that reach the distal ileum and colon undergo fermentation to short-chain fatty acids and gases, which can trigger GI symptoms^76,77^ (Figure 2). A general overview of food convenient for this diet is provided in Table 2. It is conceivable that especially FD patients might also benefit from this dietary approach, since many FODMAPs require AGAL for a proper digestion. In this respect, the classical Mediterranean, Okinawan, or anti-inflammatory diets are probably not recommendable due to the FODMAP-rich components such as legumes (beans, peas, chickpeas, soy, and lentils), whole grains, garlic, and so on. Compared to patients with IBS, after a 2–6 -week elimination phase with a FODMAP-low diet, responding FD patients should undergo a reintroduction phase to determine which FODMAP-rich components are tolerated and which are not.^77^

**Table 2.** Overview of fermentable oligo-, di-, mono-saccharides and polyol (FODMAP)-rich and -low food and recommendations

	Don’ts FODMAP-rich food	Do’s FODMAP-low food
fruits (fructose, oligosaccharides, polyols)	apples, pears, apricots, cherries, dates, lychee, blackberries, currants, water melons, plums, mangos, peaches, tinned fruits, fruit juices, dried fruits	lemons, oranges, tangerines, kiwi fruits, honey melons, pineapples, grape fruits, berries, passion fruits, papaya, rhubarb, bananas, grapes
vegetables (fructose, oligosaccharides, polyols)	asparagus, avocado, artichokes, beans, chicoree, chickpeas, peas, onions, shallots, cauliflower, garlic, pickled cabbage, lentils, mushrooms, beetroot, savoy	salads, pak choi, cucumber, mangold, okra, zucchini, aubergine, sweet pepper, tomatoes, sprouts, olives, carrots, roots, potatoes, parsnips, radish, ginger, fennel, spinach, pumpkin, nori-algae, broccoli, green runner beans
grains (oligosaccharides)	gluten-reach grains (especially barley, wheat, rye)	gluten-free grains, millet, oat bran, spelt, corn, quinoa, amaranth, psyllium husks, buckwheat, rice, tapioca (manioc)
sweeteners (fructose, polyols)	corn syrup, fructose syrup, agave syrup, honey, mannitol sugar substitutes such as maltitol, isomalt, xylitol, sorbitol	little table sugar, glucose, maple leaf syrup, sugar substitutes such as aspartame
dairy products (lactose)	lactose containing milk and yogurt, cream cheese, cream, milk powder, sour cream	lactose-free milk and yogurt, hard cheese, brie, camembert, feta, mozzarella, coconut and soy milk, butter
meat	processed, fatty, fried or breaded meat, cold meat	lean meat, chicken, Turkey, eggs, lamb
fish	processed, fatty, fried or breaded fish	seafood every fish
drinks	lemonades, fruit juices, malt coffee, black tea (long drawn), fennel tea, chamomile tea beer, wine/sparkling wine (sweet, semi-dry), liqueur, rum, sherry	mineral water, carrot juice, cranberry juice, coffee, green and white tea, black tea (short infusion), peppermint tea wine/sparkling wine (dry)
other	margarine ketchup cashews, pistachios	tofu, vinegar, olive oil, plant oil, rapeseed oil, mustard less than 15 nuts per day chocolate (dark)

In addition, dietary supplements containing high amounts of orally delivered AGAL seem to be beneficial for gastrointestinal symptoms^78,79^ and further support the nutrition hypothesis. In a randomized double-blind placebo-controlled study, the administration of orally delivered AGAL significantly reduced breath hydrogen excretion and severity of flatulence in healthy volunteers during a meal containing 420 g cooked beans.^79^ Further studies demonstrated that non-FD patients with irritable bowel symptoms due to galacto-oligosaccharide intolerance may benefit from nutritional supplements containing high amounts of recombinant AGAL enzyme, an enzyme with amylase like-activity.^48,80^

Regarding the current literature, orally delivered AGAL might influence GI symptoms in patients with FD by a combination of several factors. First, orally delivered AGAL supports a proper digestion of nutrition. Second, a potential depletion of intestinal (lyso)-Gb_3_ might result in an amelioration of proinflammatory status and less dysbiosis. Third, if orally delivered AGAL is absorbed within the gut, this might lead to a depletion of Gb_3_ in, i.e. endothelial cells and neurons. In this respect, it is suggested that within the intestine AGAL digests and breaks down complex galacto-oligosaccharides (such as raffinose, stachyose, or verbascose), a process that probably reduces bowel symptoms including abdominal bloating and diarrhea by decreasing the colonic fermentation and thus gas production.^65^ In a recent case study, it was further demonstrated that daily oral substitution with oral AGAL can significantly decrease GI symptoms (diarrhea and abdominal pain) in FD patients.^54^ It is conceivable that the observed effects in this study are mainly due to an improved digestion of the food by the contained AGAL and beta-glucosidase. Hence, many GI symptoms in patients with FD might be due to the lack of a proper galacto-oligosaccharide digestion within the intestine due to absent or reduced AGAL activity, potentially leading also to an overgrown intestinal flora.^13^ Future studies are now warranted to confirm the effect of orally delivered AGAL on GI symptoms and gut microbiota in FD patients.

Due to existing dysbiosis, probiotics, and prebiotics may also alleviate gastrointestinal symptoms in FD patients. Probiotic bacteria are living microorganisms that can reduce dysbiosis, while prebiotics, as a substrate for intestinal healthy bacteria, promote the growth of *Bifidobacteria*, among others, and thus reduce bacterial dysbiosis.^81,82^

Many probiotic bacteria are members of the gut microbiota and are included in foods to improve gut function. In this respect, the immunomodulatory ability of bacterial exopolysaccharides is reported.^83,84^ Consumption of food rich in probiotics or the supplementation of probiotics partially affected immune function by altering endogenous metabolic activities of microbiota.^85^ Interestingly (with respect to classical FD patients suffering from end-stage renal disease), probiotics were also reported to decrease pro-inflammatory cytokine levels (in blood) in patients undergoing dialysis.^86^ In addition, prebiotics such as polysaccharides from the green algae *Enteromorpha clathrataprovide* are a selective food base for intestinal bacteria such as *Lactobacilli* and *Bifidobacteria* and can specifically influence the composition of the intestinal flora,^87^ reducing dysbiosis by increasing microorganisms with a health-promoting influence in the colon.

## Hypothetic modifiers of GI symptoms in patients with FD

**Figure 3.**
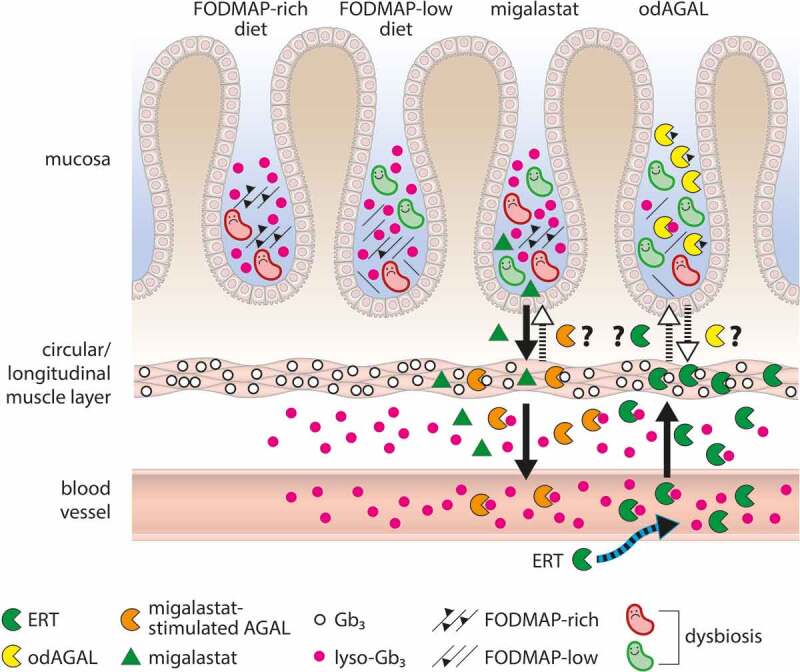
**Potential modifiers of GI symptoms in patients with FD**. Normal or FODMAP-rich nutrition will not be adequately digested within the gut, potentially due to the lack of AGAL. In addition, lyso-Gb_3_ promotes dysbiosis. A FODMAP-low diet will reduce dysbiosis, although the effect of lyso-Gb_3_ on intestinal flora is still present. Treatment with migalastat results in increased intra- and potentially extracellular AGAL activities and (lyso)-Gb_3_ depletion. Epithelial gut cells might also secrete functionally active AGAL to the gut lumen assisting in lyso-Gb_3_ decrease as well as nutrient digestion, which might result in reduced dysbiosis, but needs to be confirmed in appropriate studies. ERT results in intra- and extra-cellular (lyso)-Gb_3_ depletion. If ERT also affects lyso-Gb_3_ or nutrient digestion in the gut lumen is questionable, especially due to the nonacidic pH within the human intestine. Orally delivered (od)AGAL might assist in FODMAP-rich nutrient digestion and potentially in lyso-Gb_3_ depletion. Both could indirectly reduce dysbiosis due to the residual AGAL activity at neutral conditions, but needs further confirmation. If odAGAL will be internalized by epithelial gut cells and can deplete intra- and extra-cellular (lyso)-Gb_3_ afterward needs to be confirmed in further studies. AGAL: α-galactosidase A; ERT: enzyme replacement therapy; FODMAP: fermentable oligo-, di-, monosaccharides, and polyols; Gb_3_: globotriaosylceramide; lyso-Gb_3_: globotriaosylsphingosine; od: orally delivered.

Based on the current literature and on personal experience with affected patients, the following model of potential modifiers of GI symptoms in patients with FD could be assumed (Figure 3). Normal or FODMAP-rich nutrition will not be adequately digested within the gut, hypothetically due to the lack of functional (secreted) AGAL. In addition, lyso-Gb_3_ may promote dysbiosis. A FODMAP-low diet may reduce dysbiosis, although the effect of lyso-Gb_3_ on intestinal flora is still present. Treatment with migalastat was associated with a clearance of (lyso)-Gb_3_ deposits from several cell types, including kidney peritubular capillaries,^16^ podocytes,^88^ endothelial cells and mesangial cells,^16^ and patient-derived urinary primary cells^89^ (*in vitro*), which might also result in an improved neuronal and vascular function in the intestine. It is conceivable that epithelial gut cells might also secrete functionally active AGAL to the gut lumen assisting in lyso-Gb_3_ decrease as well as nutrient digestion, which might result in reduced dysbiosis. However, future studies need to demonstrate whether endogenous AGAL will be secreted into the gut lumen or not and if the residual AGAL activity at neutral pH values^90^ in the intestine is sufficient. ERT results in intra- and extra-cellular (lyso)-Gb_3_ depletion comparable to chaperone therapy. Whether ERT also affects lyso-Gb_3_ or nutrient digestion in the gut lumen is questionable. Orally delivered (od)AGAL assists in FODMAP-rich nutrient digestion and potentially in lyso-Gb_3_ depletion within the gut lumen, both potentially indirectly reducing dysbiosis, too. If odAGAL^54^ will be internalized by epithelial gut cells and may deplete intra- and extra-cellular (lyso)-Gb_3_ afterward needs to be confirmed in further studies.

## Conclusion

Patients with FD often suffer from GI symptoms, such as abdominal pain, diarrhea, bloating, flatulence, nausea, and vomiting, which lead to a reduced quality of life. Therefore, physicians should also consider FD as a possible differential diagnosis when clinically assessing patients with unspecific upper and lower GI symptoms. Diagnostic tools such as patient-reported GI symptom questionnaires (such as GSRS) may aid the diagnosis of FD in patients with GI symptoms of unknown cause. In patients with unspecific GI symptoms, where FD is suspected on the basis of the clinical picture, FD diagnostics should be carried out by detection of a reduced AGAL activity in the blood (man) or molecular genetic detection of a causal *GLA* mutation (woman).

Several important FD-specific mechanisms such as dysfunction of the autonomic nervous system, vasculopathy, tissue inflammation, and dysbiosis are involved in GI manifestations and disease progression. It can be supposed that nutrition plays an important role, too. In addition to a timely initiation of FD-specific therapy, affected patients with GI symptoms might benefit from a FODMAP-low diet, pro- and prebiotics, and/or a cost-effective oral substitution with AGAL to assist a proper digestion. We conclude that FD patients with unspecific GI symptoms need more attention from treating physicians.

## Data Availability

No new data were generated or analyzed in support of this research.
